# The clinical genetics of phaeochromocytoma and paraganglioma

**DOI:** 10.1590/2359-3997000000299

**Published:** 2017-09-01

**Authors:** P. T. Kavinga Gunawardane, Ashley Grossman

**Affiliations:** 1 Oxford Centre for Diabetes, Endocrinology and Metabolism University of Oxford UK Oxford Centre for Diabetes, Endocrinology and Metabolism, University of Oxford, UK; 2 Green Templeton College University of Oxford UK Green Templeton College, University of Oxford, UK

**Keywords:** Phaeochromocytoma, paraganglioma, genetics

## Abstract

Phaeochromocytoma and paraganglioma are rare catecholamine-producing tumours, recognised to have one of the richest hereditary backgrounds of all neoplasms, with germline mutations seen in approximately 30% of patients. They can be a part of genetic syndromes such as MEN 2 or Neurofibromatosis type 1, or can be found as apparently sporadic tumours. Germline mutations are almost always found in syndromic patients. Nonetheless, apparently sporadic phaeochromocytoma too show high germline mutation rates. Early detection of a genetic mutation can lead to early diagnosis of further tumours via surveillance, early treatment and better prognosis. Apart from this, the genetic profile has important relevance for tumour location and biochemical profile, and can be a useful predictor of future tumour behaviour. It also enables family screening and surveillance. Moreover, recent studies have demonstrated significant driver somatic mutations in up to 75% of all tumours. Arch Endocrinol Metab. 2017;61(5):490-500

## INTRODUCTION

Phaeochromocytomas and paragangliomas are uncommon tumours originating from the neural crest-derived chromaffin cells of the adrenal medulla and sympathetic/parasympathetic ganglia respectively. The highest prevalence of phaeochromocytoma is in the fourth and fifth decades, while its incidence is equal in men and women.

Malignant phaeochromocytoma is defined by the presence of distant metastases in non-chromaffin tissues, which only account for about 15-20% of lesions (1). Although a majority of these catecholamine secreting tumours are by definition non-malignant, most of them secrete an excess of one or more catecholamines: epinephrine (adrenaline), norepinephrine (noradrenaline) or dopamine, which gives rise to a wide array of clinical complications, including resistant hypertension, tachyarrhythmia and cardiomyopathy.

The genetic nature of these catecholamine secreting tumours has been an area of extensive research interest over the last few decades, and as a result multiple genes have been identified in association with phaeochromocytoma as well as paraganglioma. Therefore, in contrast to conventional teaching of a 10% familial tendency (“the 10% rule”), phaeochromocytoma has now been shown to have a much higher genetic tendency with more than one third of patients harbouring a disease-causing germline mutation (2). As these tumours are recognised to have one of the richest hereditary backgrounds among all neoplasms, most authorities and guidelines currently recommend genetic testing of all patients for the presence of disease-causing mutations (3).

## CLINICAL IMPLICATIONS OF GENETIC TESTING

Genetic analysis in phaeochromocytoma is an extremely useful tool in clinical practise as accumulating data have shown genetics to be equally valuable not only for screening but also for diagnosis and prognostication of hereditary phaeochromocytoma.

Firstly, differentiation between the benign and malignant nature of a phaeochromocytoma can be a challenge to the managing physician. Genetic evaluation can be of assistance in this situation, where one can predict a higher tendency towards the development of malignant disease with metastases in patients harbouring certain mutations (e.g. mutations of *SDHB* lead to metastatic disease in 40% or more of affected patients, or less commonly seen *MAX* and *FH* mutations) (4,5). In fact, germline genetic forms of phaeochromocytoma are often multiple, extra-adrenal and recurrent; consequently, regular surveillance and strict follow-up is recommended for better prognosis of such patients. Secondly, establishing certain hereditary syndromes with associated tumours with a high malignant potential (e.g. patients with MEN 2 – 100% potential to develop medullary carcinoma of thyroid) can lead to early diagnosis and treatment of other malignant syndromic manifestations in patients and relatives. Finally, identification of germline mutations of phaeochromocytoma can lead to early diagnosis and treatment, offering better prognosis to family members via screening and surveillance.

## PATHOGENESIS: GENETIC GERMLINE HETEROGENEITY

The pathogenesis of the hereditary nature of phaeochromocytoma can be described in two main clusters (6). The first cluster contains pseudohypoxia-driven tumours including *VHL*, *SDH*, *EGLN1* and *HIF2A* mutant tumours. The second cluster contained the kinase signalling subgroup including the *RET*, *NF1*, *TMEM 127* and *MAX* mutant tumours.

The feature common to all cluster 1 tumours is the activation of HIFs. Hypoxia inducible factors (HIFs) are transcription factors induced as a physiological response to cellular hypoxia. In the presence of *VHL*, *SDH*, *EGLN1* and *HIF2A* mutations, HIFs are induced and stabilised, pointing the cell towards a pseudo-hypoxic state. Pseudohypoxia occurs when HIF pathways are constitutively activated, regardless of oxygen levels. This cellular pseudohypoxia leads to epigenetic modifications in HIF target genes affecting multiple cellular processes including apoptosis, angiogenesis, proliferation, migration, and invasion.

The second cluster of genes cause catecholamine secreting tumours by way of affecting the kinase signalling pathways. Activation of *RET* proto-oncogene in MEN 2 and inactivation of *NF1* leads to activation of RAS/MAPK and PI3/AKT signalling pathways. Similarly, *TMEM127* mutation activates the mTOR pathway while *MAX* mutation too has been established to affect the downstream mTOR pathway via the MYC-MAX- MXD1 network.

However, the pathogenesis of phaeochromocytoma may not be quite as simple, where there can be significant overlap due to high degree of redundancy and cross-talk between constituents of these pathways. For example, mTOR can activate HIF, while MYC cooperates with HIF2α in oncogenesis (6). Furthermore, there is increasing evidence that *SDH* and related mutations can lead to the build-up of succinate which can act as an oncometabolite causing marked changing in patterns of gene methylation.

## FAMILIAL SYNDROMES ASSOCIATED WITH PHAEOCHROMOCYTOMA/PARAGANGLIOMA

### Multiple endocrine neoplasia-2 (MEN 2)

Multiple endocrine neoplasia-2 is one of the earliest syndromes to have been associated with phaeochromocytoma and is caused by an activating (gain-of-function) germline mutation in the *RET* proto-oncogene located on chromosome 10q11.2. This proto oncogene encodes a transmembrane receptor tyrosine kinase involved in the regulation of cell proliferation and apoptosis (7).

Sipple first described an association between thyroid cancer and phaeochromocytoma in 1961 and since then this familial constellation of pathology has been studied extensively, including the identification of the underlying germline mutation. Clinically, there are three main subtypes of MEN 2; 1) MEN2A is characterised by medullary thyroid cancer in 95% of patients, phaeochromocytoma in 40-50% and primary hyperparathyroidism in 20%-30%; 2) MEN2B accounts for approximately 5% of MEN syndromes and has medullary thyroid cancer in 100%, phaeochromocytoma in 50% of cases, a Marfanoid body habitus, and multiple mucosal ganglioneuromas; however, it is *not* associated with hyperparathyroidism. 3); the third group is the rarest *RET* proto-oncogene associated MEN2 which represents familial medullary thyroid cancer alone (8,9). Identification of phaeochromocytoma is vital in these patients with MEN2 to avoid perioperative hypertensive crisis during thyroidectomy for medullary thyroid carcinoma.

The genetic defect in MEN 2 is inherited in an autosomal dominant pattern with high penetrance. In MEN 2, clinical heterogeneity has been noted due to mutations in several codons in the *RET* gene: the great majority of MEN 2A (now changed simply to MEN2) are associated with a mutation at codon 634, exon11 which codes for the extra-cellular domain of RET, while for MEN 2B (now MEN^3^) the dominant mutation lies in codon 918, exon 16 which codes for part of the intra-cellular domain. In MEN 2A the *RET* mutation occur in the extracellular domain of the RET and causes ligand-independent activation of PI3K–AKT, RAS, p38 MAPK and JUN N-terminal kinase pathways, resulting in the stimulation of cell growth, differentiation and survival. On the other hand, MEN2B-related mutations target a few codons affecting the catalytic site of the kinase, leading to loss of substrate specificity only. Therefore, it has been established that the subtle changes in the clinical presentation and molecular outcome is due to these genetic variations in the mutations (10).

Phaeochromocytomas seen in MEN 2 are frequently bilateral, adrenal in localisation and almost always benign (11) with the rate of malignant transformation being between 1 to 5%. However, it has been reported that children with phaeochromocytoma diagnosed with MEN2B have a higher risk of harbouring a malignant phaeochromocytoma compared to children with MEN2A or sporadic phaeochromocytoma (12).

The biochemical phenotype is also rather different in patients with phaeochromocytoma associated with MEN2. They commonly overexpress phenylethanolamine N-methyltransferase which is an enzyme that converts norepinephrine to epinephrine, leading to hypersecretion of epinephrine in large amounts. This is consistent with increased levels of metanephrine, which is a catecholamine O-methylated metabolite of epinephrine, detected in plasma and excreted in urine in these patients (13). Interestingly, only half of the patients with MEN2A harbouring a phaeochromocytoma present with it, which might be explained by earlier presentation with medullary carcinoma of the thyroid or family screening (8).

Genetic identification is also important as children born with the codon 634 mutation are advised to undergo total thyroidectomy before the age of 5 years, while with 918 mutations thyroidectomy in the first year is recommended.

With other mutations, it is suggested that the specific published data on such families are explored for prognosis and therapeutic options.

## NEUROFIBROMATOSIS TYPE 1 (NF1)

NF1 or von Recklinghausen’s disease is another autosomal dominant disorder, characterized by neurofibromas, *café-au-lait* spots, freckling, Lisch nodules, phaeochromocytoma and paraganglioma: 0.1 to 5.7% of patients with the *NF1* gene present with solitary and benign phaeochromocytomas (14).

NF1 is due to an inactivating mutation in the tumour suppressor gene *NF1*, located on chromosome 17q11.2. The *NF1* gene encodes a large, 327 kDa protein called neurofibromin, belonging to a family of GTPase-activating proteins (GAP). This protein downregulates a cellular proto-oncogene, p21-RAS. RAS is a major oncogene in human malignancies. It is well known to regulate cell growth and differentiation, and activates a number of signalling pathways including the stem cell factor, mTOR, and MAP kinase pathways. mTOR is a crucial downstream signal of both RAS and RET pathways, and is aberrantly activated in NF1-deficient malignant peripheral nerve sheath tumours, phaeochromocytomas and paragangliomas (15).

Fifty per cent of phaeochromocytomas in NF1 are familial while the rest are due to *de novo* mutations (16). Familial NF1 shows “complete penetrance”, where the individual carrying the mutation will be almost always affected by it. However, it is highly variable in its “expression”, indicating that the severity of disease of the affected individuals can vary marked within families (17). Since the cloning of the *NF1* gene in 1990, numerous constitutional mutations of patients have been described (Upadhyaya and Cooper 1998, NNFF International NF^1^ Genetic Mutation Analysis Consortium, Human Gene Mutation Database Cardiff) including cytogenetically visible translocations, intronic rearrangements affecting splicing, deletions, duplications, insertions; and many different point mutations and substitutions (18). Although many mutations have been identified in association with NF1 there is still no conclusive evidence to correlate the genotype with the phenotype or predict clinical risk factors with certain mutations (19).

The diagnosis of NF1 is based on multiple cutaneous and bony lesions (Table 1).

**Table 1:** NIH diagnostic criteria for neurofibromatosis type 1 (20)

Two or more of the following clinical features must be present:
Six or more *café-au-lait* macules of more than 5 mm in greatest diameter in pre-pubertal individuals, and more than 15 mm in greatest diameter in post-pubertal individuals
Two or more neurofibromas of any type or one plexiform neurofibroma
Freckling in the axillary or inguinal regions
Optic glioma
Two or more iris hamartomata (Lisch nodules)
Distinctive bony lesion such as sphenoid dysplasia, or thinning of the long bone cortex with or without pseudo-arthrosis
A first-degree relative (parent, sibling, or offspring) with NF1 based on the above criteria

Patients with NF1 have an increased frequency to develop both benign and malignant tumours. Optic path gliomas are the predominant type of central nervous system tumours. Patients can also develop astrocytomas, brain stem gliomas, insulinomas and soft tissue sarcomas.

Phaeochromocytoma is a rare but important manifestation of NF1 which usually presents in fourth or fifth decade, by which time most patient would have developed some form of a cutaneous manifestation of NF1. Mostly phaeochromocytoma in NF1 are benign and unilateral; however, they can occasionally be bilateral or extra-adrenal and up to 12% of these phaeochromocytomas can be malignant (14,21).

## VON HIPPEL-LINDAU (VHL) SYNDROME

VHL is a rare (incidence of 1:36,000 in the general population) autosomal-dominant inherited syndrome associated with the development of a variety of benign and malignant tumours.

Families and individuals with VHL have been divided into types 1 and 2, based on their likelihood of developing phaeochromocytoma. Patients with type 1 VHL have a low likelihood of developing phaeochromocytoma, although they are at a higher risk of developing other VHL-associated tumours. Families with type 2 disease are at an increased risk of developing phaeochromocytoma. Type 2 is again divided into 3 groups: 2A phaeochromocytoma with low incidence of renal cell carcinoma (RCC), 2B phaeochromocytoma with high incidence of RCC, 2C only develop phaeochromocytoma as apparent sporadic tumours. These sub classifications are used as a guide and are not by any means absolute. In general, mutations which lead to complete loss of function tend not to be associated with phaeochromocytomas.

VHL-related lesions occur at a wide range of ages with the retinal lesions commencing at a very young age. Patients need to be screened for CNS haemangioblastoma, retinal angioma, clear cell renal cell carcinoma, pancreatic neuroendocrine tumours (which are seen in around 10%) and middle ear tumours regularly. Haemangioblastomas are the most common lesions associated with VHL disease, affecting 60 to 84%, typically occurring in the cerebellum or spinal cord (22). The incidence of development retinal capillary haemangioblastomas increases with age where 70% of VHL patients will harbour multifocal, bilateral lesions by the age of 60 (23). Almost all RCC in VHL are clear cell carcinoma with a mean age of onset of 44 years.

VHL is caused by a heterozygous germline mutation on the VHL tumour suppressor gene on chromosome 3p25.5 and contains three exons. The *VHL* gene encodes two proteins, pVHL_30_, pVHL_19_. They are “substrate recognition components” which target HIF1α and HIF2α for proteasomal-mediated degradation. Therefore, loss of function of VHL leads to inappropriate accumulation of HIF and subsequent activation of the hypoxic response, promoting angiogenesis, glycolysis and proliferation. This explains the predisposition for patients to develop vascular and other types of tumours in VHL syndrome (24).

In VHL syndrome, catecholamine-secreting tumours develop in 10-20% with a mean age of presentation of 30 years (25). They are more frequently benign, intra-adrenal and bilateral. However, rarely mediastinal, abdominal and pelvic sympathetic paragangliomas as well as head and neck parasympathetic paragangliomas have also been reported (26).

Interestingly, patients harbouring the *VHL* mutation have a lower incidence of hypertension and have specifically elevated normetanephrine, in contrast to patients with MEN-2 and NF, who show elevated metanephrine levels (13,27).

## FAMILIAL CATECHOLAMINE-HYPERSECRETING TUMOURS IN SUCCINATE DEHYDROGENASE (SDH) GENE MUTATION

SDH is an enzyme complex on the inner mitochondrial membrane with 4 subunits, SDHA, SDHB, SDHC, and SDHD. This enzyme complex catalyses the important oxidation of succinate to fumarate in the Kreb cycle with the reduction of ubiquinone to ubiquinol via the mitochondrial respiratory chain.

The four subunits of the enzyme complex are encoded by four *SDH* genes – *SDHA*, *SDHB*, *SDHC*, and *SDHD*. SDHA and SDHB are the hydrophilic subunits responsible for the catalytic process of the SDH enzyme complex. SDHA is a flavoprotein and SDHB is an iron-sulphur protein. SDHC and SDHD, on the other hand, are hydrophobic and act as the two anchorage proteins. Apart from these four proteins a fifth factor, succinate dehydrogenase complex-assembly factor 2 (SDHAF2), essential for the proper function of the SDHA subunit (cofactor of flavin adenine dinucleotide), has now been recognised. *SDHAF2* is encoded by SDHAF2 gene which, similar to genetic defects in other SDH gene defects can cause familial catecholamine-hypersecreting tumours. Apart from catecholamine secreting tumours, genetic defects in the SDH complex less frequently gives rise to renal cell carcinomas and gastro- intestinal stromal tumours (GISTs), and more recently to pituitary adenomas (28-31).

The two main functions of SDH are the oxidative dehydrogenation of succinate to fumarate in the tricarboxylic acid cycle (TCA) cycle and the reduction of ubiquinone in the electron transport chain during ATP synthesis. Therefore, the SDH enzyme complex plays a vital role in the initial deprotonation step, where electrons are derived from succinate oxidation via FAD. After the electrons have been liberated from the oxidation of succinate, they are tunnelled along the Fe-S relay to an awaiting ubiquinone molecule.

The common feature in all *SDH* mutations is the inactivation of the SDH complex which leads to the accumulation of succinate and increase in oxygen free radical production. Succinate affects HIF stability through its effects on post-translational regulation of HIFα subunits, an essential step for the recognition of HIF for proteasome-mediated degradation. Therefore, accumulation of succinate and an increase in oxygen free radical production in SDH inactivation leads to stabilisation of HIF.

Through similar mechanisms as in VHL, stabilisation of HIF-α activate multiple hypoxia-dependent pathways leads to epigenetic modifications in HIF target genes (DNA and histone hypermethylation). These genes that are affected by hypermethylation have been implicated in many vital effects on cellular processes including apoptosis, angiogenesis, energy metabolism, proliferation, migration, and invasion of tumour cells (32). Thus, HIF-α stabilisation in *SDH* mutations cause subsequent epigenetic modifications giving rise to multiple benign and malignant tumour pathology including phaeochromocytomas and paragangliomas.

Interestingly, both DNA demethylation and histone demethylation associated with an *SDH* mutation can be corrected by the addition of the methylase inhibitor, decitabine. These findings support a potential reversible hypermethylation process in patients with an SDH mutation, suggesting a possible therapeutic pathway. Moreover, over the last several years, new molecules to inhibit HIF2α have been developed, especially in the treatment of clear cell carcinoma of the kidney (33). PT2385 is one such molecule and it binds to a HIF-2α unique protein pocket in the PAS-B domain, and thus, prevents the HIF-2α-ARNT dimerization and the formation of an active HIF-2 transcription complex. The development of these molecules (PT2385 and PT2399) have may provide a therapeutic opportunity to perhaps successfully treat pharmacologically several types of cancers which currently have limited therapeutic options (e.g. patients with SDHB-related metastatic phaeochromocytoma/paraganglioma) (34). In addition, previous evidence suggested that SDH-deficient cells rely on lactate dehydrogenase A (LDHA) for regeneration of NAD+ or pyruvate carboxylase for the uptake of extracellular pyruvate and increased aspartate synthesis, both raising the possibility that LDH inhibition might be selectively toxic to SDH-loss cells. Development of these molecules may give the possibility of non-cytotoxic metabolite for the treatment of SDH-loss in phaeochromocytoma/ paragangliomas.

## *SDHD* MUTATION

Fifteen percent of phaeochromocytoma and paraganglioma are associated with germline *SDH* mutations. Inactivating mutations in the *SDHD* gene, autosomal-dominantly acquired, give rise to familial parasympathetic head and neck paragangliomas. They can also give rise to sympathetic extra-adrenal paragangliomas and rarely unilateral phaeochromocytoma. The head-and-neck paragangliomas are usually bilateral or multifocal. Although, the paragangliomas can be recurrent they are rarely malignant (< 5%) (35). Intriguingly, *SDHD* mutations are highly penetrant and show maternal genomic imprinting (36). Thus, almost all tumours are only seen in the children of male-affected parents, and the mutation is inactivated if inherited from the maternal side (although it will still be genetically transmitted).

## *SDHB* MUTATION

Germline mutations of SDHB gene are inherited as autosomal dominant with the presence of sympathetic extra-adrenal paragangliomas, followed by adrenal phaeochromocytomas and parasympathetic head and neck paragangliomas (37,38). Typically, they originate from extra-adrenal locations in the abdomen, thorax and the pelvis and are usually solitary tumours with a significantly high malignant potential (30%) (4,38). Therefore, all patients with metastatic phaeochromocytoma or paragangliomas should undergo *SDHB* mutation testing at the very least.

The typical age of presentation of paragangliomas due to *SDHB* mutations is 30 years. However, they can present at any age, including in childhood. Moreover, an *SDHB* mutation has a poor genotype and phenotype correlation due to low penetrance and high variable expression, where even identical mutations give rise to different types of tumours in location, behaviour and severity (4). The predominant biochemical phenotype of an *SDHB* mutation is hypersecretion of dopamine alone or dopamine and norepinephrine. Therefore, increased levels of 3-methoxytyramine, which is a product of dopamine metabolism, could help biochemically identify SDHB or other likely malignant tumours (13). Apart from biochemistry, immunohistochemistry for SDHB too can aid in the discrimination between SDHB and other mutations. If the phaeochromocytoma or the paraganglioma is due to an *SDHB* mutation, staining the tumour for SDHB will be negative with a sensitivity of 100% and specificity of 84% for *any* type of *SDH* mutation (39).

## *SDHC* MUTATION

*SDHC* mutation is located on chromosome 1q21 and is similarly inherited in an autosomal dominant pattern. However, it is rare and gives rise to benign head-and-neck paragangliomas as well as sympathetic paragangliomas and phaeochromocytomas; these can be multiple (40).

## *SDHAF2* MUTATION

Inactivating mutations in the SDHAF2 gene has recently been recognised to cause a rare type of familial paraganglioma syndrome which causes head-and-neck paragangliomas, exclusively in children of fathers carrying the defective gene. This point towards a maternal imprinting and is inherited in an autosomal dominant manner. The mean age of presentation is 30 years and studies suggest that screening for SDHAF2 is important in patients with head-and-neck paragangliomas with either a family history of head-and-neck paraganglioma, young age of onset or multiple tumours in whom *SDHB*, *SDHC*, and *SDHD* gene testing was negative (36).

## *SDHA* MUTATION

*SDHA* gene mutation was initially thought to cause Leigh syndrome, a neurodegenerative syndrome associated with subacute necrotising encephalomyelopathy with developmental delay and psychomotor regression. However, recently germ-line mutations in *SDHA* were detected in patients with both sympathetic and parasympathetic paragangliomas (41).

## OTHER GENES RELATED TO PHAEOCHROMOCYTOMAS AND PARAGANGLIOMAS

### TMEM 127

*TMEM127* is a tumour suppressor gene (four exons, chromosome 2q11) linked with mTOR (mammalian target of rapamycin) kinase pathway which has recently been associated with the development of phaeochromocytoma. Since the original report, more than 30 mutations have been identified in *TMEM127*. Although all variants were detected in germline DNA, less than 20% of patients carrying a *TMEM127* mutation report a family history of phaeochromocytomas, suggesting low penetrance of the mutant alleles (42). *TMEM127* encodes for a transmembrane protein which localizes to the plasma membrane and multiple components of the endosome machinery, including early, late and recycling endosome, Golgi complex and lysosome. Once mutated, it is mostly located in the cytoplasm, suggesting the localization of TMEM127 to endomembrane pools is important for its tumour suppressor function (42).

### >MAX Mutation

The *MAX* gene is located on chromosome 14q23 and encodes for MAX protein. MAX is a low abundance basic helix–loop–helix (bHLH) leucine zipper domain-containing protein that is predominantly found in complex with the MYC transcription factor. MYC is a common oncogene in many human cancers and MYC–MAX heterodimers bind to E-box sequences in the promoters that binds to genes encoding proteins with a wide range of cellular functions, including metabolism, growth and angiogenesis (43). Moreover, MAX can bind to other transcription factors such as MXD1, MNT and MGA which can repress the transcription of target genes, ultimately leading to the inhibition of cell growth and promotion of terminal differentiation (44). Therefore, MAX can function as both a suppressor and activator of genes involved in many oncogenic pathways. Thus, a balance between MAX complexes with MYC and MAX complexes with MYC repressors dictates the output of transcription of E box-containing genes as a result of either activation or repression (43).

Although the mechanism in which a *MAX* mutation causes phaeochromocytoma remains unclear, recent studies show that partial deletion and reintroduction of MAX results in cell growth arrest supporting the role of MAX repressing the oncogenic effects of MYC on paraganglial cells (44).

MAX associated catecholamine-secreting tumours can be either adrenal or extra-adrenal. Adrenal tumours are often bilateral (67%) with a possible association with malignant behaviour. Therefore, mutations in the *MAX* gene should be sought in patients with familial, bilateral or apparently sporadic phaeochromocytoma/paraganglioma (45).

## OTHER GENES

The actual mutation load of individual phaeochromocytomas and paragangliomas is unknown. Multiple novel germline mutations have been associated with the development of phaeochromocytoma. A few well recognized ones are *HIF2A* (also known as *EPAS1*), *KIF1B* and *EGLN1*.

KIF1B is a rare germline mutation which causes phaeochromocytoma and neuroblastoma. Located on chromosome 1p36.22, KIF1B belongs to the kinesin family encoding a protein that induces apoptosis. KIF1B acts in a prolyl hydroxylase domain‐containing protein-3 (PHD3) dependent apoptosis pathway that occurs physiologically in sympathetic lineage precursor cells during development (46).

Another rare germline mutation causing phaeochromocytoma together with congenital erythrocytosis is the *EGLN1* mutation. *EGLN1* (egl-nine-homolog-1) gene, also termed PHD2, is located on chromosome 1q42.1, encodes a prolyl hydroxylase, which has a crucial function in the oxygen-dependent proline hydroxylation of the HIF-α pathway. Therefore, through similar pseudohypoxic mechanisms as in SDH, *EGLN1* mutations can give rise to familial paraganglioma (47).

Loss of function of fumarate hydratase (FH), which catalyses the conversion of fumarate to malate, has been demonstrated to cause accumulation of the precursor metabolite, fumarate, Fumarate shares structural similarities with succinate. Similar to succinate accumulation in *SDH* mutations, fumarate accumulation in FH activates the pseudo-hypoxia driven pathways to give rise to catecholamine secreting tumours (48).

Similar to succinate and fumarate accumulation, which leads to enzymatic inhibition of multiple α-KG-dependent dioxygenases in the Krebs cycle, a new germline mutation in *MDH2* (malate dehydrogenase 2) has been found to cause phaeochromocytoma/paraganglioma (with possible metastasis). This mutation causes a deletion in the tumour suppressor gene prompting a stable silencing of MDH2 expression. It has been suggested that suppression of MDH2 leads to accumulation of malate which, similar to succinate, inhibits the HIFα pathway. This mutation was found in patients neuroblastomas, as well as in malignant phaeochromocytoma and paraganglioma (49).

Mechanisms underlying phaeochromocytoma are astonishingly diverse, with both inherited and somatic drivers influencing tumorigenesis through a broad range of biological pathways. Apart from germline mutations, recent studies have attempted to locate somatic mutations in the phaeochromocytoma/paragangliomas.

Somatic mutations of the *HRAS* gene, which is one of the most frequently disordered genes in many malignancies was isolated in phaeochromocytoma by exome sequencing (50). These mutations target the signal downstream of the RAS-MAPK pathway. Identification of somatic mutations is useful specially in the differentiation between malignant and benign phaeochromocytoma, which can be quite challenging to the managing physician.

Another well-known somatic mutation is the *HIF2*α mutation. This somatic gain-of-function mutation targets the HIF2α-stabilising prolyl sites, Pro531, affecting the conformation of HIF2α. This conformational change induces downstream targets leading to tumour growth. Interestingly, despite the somatic nature, patients with HIF2α mutation were found to develop somatostatinomas and 50% developed early onset or congenital polycythaemia. It seems probable that this is due to germline mosaicism (51). Interestingly, ophthalmic complications are also being recognised in this syndrome.

Recent data have revealed that DNA translocation and fusion genes act as a component of phaeochromocytoma tumorigenesis. Moreover, certain germ-line mutations as well as somatic mutations and fusion genes can be used as markers/predictors of aggressive disease-free survival (ADFS), the time until the occurrence of distant metastases, local recurrence, or positive regional lymph nodes. Apart from germ-line mutations such as *SDHB*, certain somatic mutations including *ATRX* and *MAML3* fusion gene were shown to predict clinical outcome in patient with phaeochromocytoma (52). Certain of these gene products seem to be involved in the b-catenin pathway, indicating a separate sub-group of this type of tumour. Currently around 75% of all phaeochromocytomas and paragangliomas show either a clear germline or a likely somatic driver mutation.

Finally, deep exome-sequencing studies have revealed very low frequency germline gain-of-function mutations in histone methylators such as *H3F3A* and *H3K9*; this is an area of intense research and undoubtedly more will be learnt with clinical applicability in the near future.

## APPROACH TO GENETIC TESTING IN CLINICAL PRACTICE

**Figure 1:**
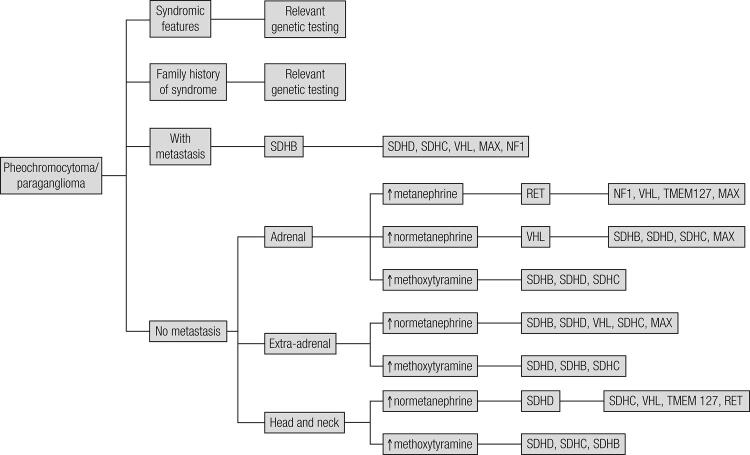
Decisional flow-chart for genetic testing in patients with a proven phaeochromocytoma/paraganglioma.

With the rising number of genes identified in association with phaeochromocytoma/paraganglioma, routine testing for all known germ-line has in the past been expensive and time-consuming. It is also important to remember that the majority of these tumours are still sporadic and may not carry a germline mutation. Therefore, the suggestion has been to employ various predictors to suggest a screening process for genetic testing: based on many studies, germ-line mutations are common in patients with early onset disease (< 45 years), bilateral phaeochromocytoma, extra-adrenal disease (e.g. head and neck paraganglioma), multifocal, recurrent or malignant disease and a positive family history of phaeochromocytoma. Therefore, patients with these features were considered for genetic testing (25,53). Then, depending on certain feature associated with different mutations one could decide on the order of genes to be tested (Figure 1). This decision-making process could be guided by several other factors including presence of syndromic clinical features on clinical evaluation, positive family history of syndromic features (e.g. a family member with medullary thyroid carcinoma suggest possible MEN2), tumour location, type of catecholamine produced by the tumour and histological evaluation.

For patients with sporadic phaeochromocytoma (without family history or syndromic feature), decision making can be aided by several tumour characteristics such as tumour location, biochemical phenotype and histopathology. A summary of the indicative factors is given below:

## LOCATION OF THE TUMOUR

Considering the location of the tumour; intra-adrenal tumours suggest possible *RET*, *VHL*, *NF1*, *TMEM 127*, *MAX* or rarely *KIF1B* mutations. In addition, bilateral phaeochromocytomas are mostly found with these same mutations (11,12,14,21,26,42,45,53). On the other hand, *SDH* mutations cause intra-adrenal tumours less commonly; 25% of *SDHB*-related tumours are phaeochromocytomas while the frequency of intra-adrenal tumours in *SDHD, SDHA* and *SDHC* are even lower (4,7,36,37,41,53).

Most of the extra-adrenal tumours are due to mutations in *SDH* genes (4,7,36,37,40,53). Apart from which, extra-adrenal tumours were also found in rare *EGLN1* mutation (47). Although, rare extra adrenal tumours can also be found in *VHL*, *TMEM 127*, *NF1*, and *RET* mutations as well (11,12,14,21,26,42,53).

Of the extra-adrenal tumours, head-and-neck paragangliomas hold a special importance as they have a high possibility of carrying an underlying genetic mutation. Of the *SDH* mutations, *SDHD*-related tumours are commonly seen in the head and neck region and are usually multiple. Head and neck tumour are also seen in *SDHB* and *SDHC* mutations; however, they are much less common (7,31,53). An even rarer cause for head-and-neck paraganglioma is the *SDHAF2* mutation, which should be considered if *SDHD, SDHB,* and *SDHC* testing is negative (36). Due to high rate of an underlying genetic defects, head and neck paraganglioma negative for all *SDH* mutations can be tested for *VHL* and *TMEM127* (although the possibility is very rare). Sympathetic paragangliomas, which are large, solitary tumours located in abdomen, thorax and pelvis, are often due to *SDHB* mutations, while *SDHC* and *SDHA* can rarely be causal (7,31,36,38,40,41,53).

## BIOCHEMICAL PHENOTYPE

Metabologenomics is another area that can also shed some light on the underlying genetic defect. Depending on the mutation, tumours show distinct differences in metabolic pathways that relate to or even directly impact clinical presentation. Therefore, the biochemical phenotype can be an important tool when deciding on the order of genetic testing in patients. Patient with catecholamine-secreting tumours due to *RET* and *NF1* mutations secrete high levels of metanephrine, indicating epinephrine production in the tumour, while patients with mutations in the VHL gene exhibited an increased production of normetanephrine, indicating norepinephrine production. On the other hand, *SDH* mutations, especially *SDHB* and *SDHD* mutations, frequently show elevated levels of methoxytyramine (an indicator of dopamine production and often malignancy) (13).

## HISTOPATHOLOGICAL DIFFERENTIATION

Finally, histopathological differentiation can be a useful tool when planning genetic screening in phaeochromocytoma. The presence of malignant features can suggest certain genetic defects; phaeochromocytomas and especially extra-adrenal paragangliomas of malignant nature are associated mostly with *SDHB* mutations (in 30% patients) (4,36-38,40). Malignant phaeochromocytomas can also be not infrequently seen with several mutations including *MAX* (25%) and *NF1* (12%) mutations (7,14,21,45,53). Malignant tumours are rare (< 5%) in *RET, VHL, SDHD, SDHC, SDHAF2* and *TMEM127* mutations. Immunohistochemistry can add to this by negative staining in *SDHB* and *SDHA* mutations (39,41).

## GENE PANEL SCREENING

Most recently, it has become clear that with the large number of possible genetic disturbances, simple algorithmic screening has become slow and resource intensive, and a number of groups have shown the utility of simultaneous screening for a whole panel of genes, independent of any other background information (except where there is clear evidence of a patient’s syndromic or family diagnosis). Such panel screening was initially with Sanger sequencing, and indeed using this approach we identified germline mutations in a series of patients with phaeochromocytomas in 25% of patients, including 15% of patients with unilateral sporadic non-recurrent phaeochromocytomas (54). Similarly, Brito and cols. in a meta-analysis identified germline mutations in 5% of gene-panelled sporadic unilateral tumours (55). With next-generation sequencing (NGS), this approach should probably be the assessment of choice in *all* patients presenting with phaeochromocytomas and paragangliomas (56).

## FINAL REMARKS

Phaeochromocytomas and paragangliomas have been paradigm shifters in genetic studies, being the first human tumour model recognised to carry a genetic defect in a metabolic enzyme (SDHD) two decades ago. Since then numerous genetic and epigenetic changes have been discovered in association with these tumours, opening up novel avenues for early and correct diagnosis, appropriate treatment and better prognosis for patients. These discoveries benefit not only the patient but also family members as positive genetic screening can lead to early diagnosis through regular surveillance. In conclusion, the era of NGS has opened up new avenues of rapid and successful diagnosis and effective screening.
